# Determining the 3D genome structure of a single mammalian cell with Dip-C

**DOI:** 10.1016/j.xpro.2021.100622

**Published:** 2021-06-18

**Authors:** Longzhi Tan

**Affiliations:** 1 Department of Bioengineering, Stanford University, Stanford, CA 94305, USA

**Keywords:** Bioinformatics, Sequence analysis, Cell isolation, Single Cell, Flow Cytometry/Mass Cytometry, Genetics, Genomics, Sequencing, Molecular Biology, Neuroscience

## Abstract

3D genome structure is highly heterogeneous among single cells and contributes to cellular functions. Our single-cell chromatin conformation capture (3C/Hi-C) technique, Dip-C, enables high-resolution (20 kb or ∼100 nm) 3D genome structure determination from single human and mouse cells. Dip-C is robust, fast, cheap, and does not require specialized equipment. This protocol describes using human and mouse brain samples to perform Dip-C, which has also been applied to other tissue types including the human blood and mouse eye, nose, and embryo.

For complete details on the use and execution of this protocol, please refer to Tan et al. (2021).

## Highlights

• High-resolution chromatin contact map and 3D genome structure from a single cell
• Robust, fast, and low-cost protocol without the need for specialized equipment
• Easy isolation, fixation, and analysis of nuclei from diverse human and mouse tissues
• Software packages to analyze 3D genome structure types behind transcriptional cell types

## Graphical Abstract

**Figure:**
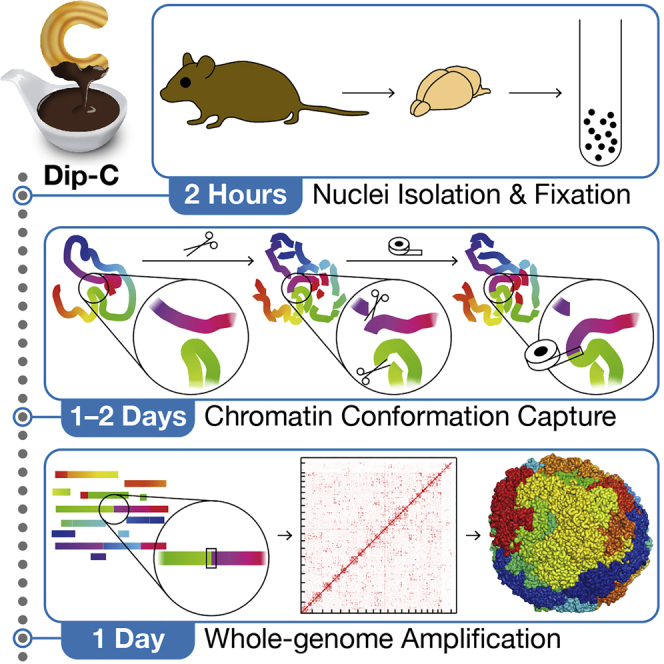

## Before you begin

The protocol below describes the application of diploid chromatin conformation capture (Dip-C) to single nuclei isolated from the human (fresh frozen postmortem) or mouse brain. Procedures include the isolation and fixation of nuclei from tissues, chromatin conformation capture (3C/Hi-C), flow-sorting and lysis of single nuclei, whole-genome amplification (WGA) with Nextera, and DNA sequencing.

If whole cells instead of nuclei are used, please follow the **Optional** instructions throughout the protocol for steps to add and to skip.

Although Dip-C is a single-cell assay, the chromatin conformation capture (3C/Hi-C) step is performed in bulk—typically on 500 k–1 m cells or nuclei. There is no lower bound on the number of cells or nuclei in principle. Users have reported success with as few as 50 cells; similar procedures have been demonstrated on as few as 1 cell (Flyamer et al., 2017; Stevens et al., 2017). For low cell numbers, the 3C/Hi-C reaction may be scaled down (fewer cells would consume less enzymes); alternative versions that do not involve centrifugation between digestion and ligation (e.g., Rao et al., 2014 or the Arima-SC kit) may further help to minimize cell loss.

If many chromatin contacts per cell are preferred, multiplex end-tagging amplification (META) (Xing et al., 2021) can be used in place of Nextera for whole-genome amplification (WGA). META detects 2 times as many contacts per cell as Nextera. Please refer to Tan et al. (2018) for detailed procedures. Note that META requires additional considerations: (a) META involves custom Tn5 transposomes and 2 additional primer removal and PCR steps. (b) During Illumina sequencing, the first 39 bp of Read 1 and of Read 2 are META transposon DNA, effectively truncating the (usable) read length. In addition, these bps have low diversity, requiring pooling with a non-META library (e.g., 20% Phi-X). (c) To sequence a library to saturation, META requires at least 2 times the sequencing depth per cell as Nextera, presenting a trade-off between the number of cells and the number of contacts per cell.

## Key resources table

**Table:** 

REAGENT or RESOURCE	SOURCE	IDENTIFIER
**Chemicals, peptides, and recombinant proteins**		

Sucrose (only necessary for nuclei)	Sigma	84097
2 M KCl (only necessary for nuclei)	Thermo Fisher	AM9640G
1 M HEPES pH 7.5 (only necessary for nuclei)	Thermo Fisher	15630080
1 M MgCl_2_	Thermo Fisher	AM9530G
IGEPAL CA-630 (only necessary for cells)	Sigma	I8896
QIAGEN Protease	QIAGEN	19155
DAPI	Thermo Fisher	D1306
TE	Thermo Fisher	AM9849
1 M DTT	Sigma	646563
10% Triton X-100	Sigma	93443
32% PFA	EMS	15714
BSA	Gemini	700-106P
PBS	Thermo Fisher	10010023
Trypan Blue (optional)	Thermo Fisher	15250061
1 M Tris pH 8.0	Thermo Fisher	AM9855G
5 M NaCl	Thermo Fisher	AM9760G
Protease inhibitor (only necessary for cells)	Sigma	P8340
10% SDS	Sigma	71736
Restriction enzyme and buffer	NEB	R0147M and B7002S (or R0125L, R0543M)
10 X T4 DNA ligase buffer	NEB	B0202S
20 mg/mL BSA	NEB	B9000S
1 U/μL T4 DNA ligase	Thermo Fisher	15224-025
0.8 U/μL Proteinase K	NEB	P8107S
0.5 M EDTA	Thermo Fisher	AM9260G
50% PEG 8000	Hampton Research	HR2-535
1 M TAPS pH 8.5	Boston BioProducts	BB-2375
Nextera Tn5 transposome	Illumina	20034197
100 ng/μL HeLa gDNA (optional)	NEB	N4006S
2 U/μL Q5 DNA Polymerase	NEB	M0491S
10 mM (each) dNTP mix	NEB	N0447S
SPRIselect beads	Beckman Coulter	B23317

**Biological samples**		

Fresh frozen postmortem human brain samples	NIH NeuroBioBank	(Xing et al., 2021)
Mouse brain samples	JAX Mice	(Tan et al., 2021); age ranged from 1 day to 1 year; both sexes were used

**Critical commercial assays**		

Disposable hemocytometer	INCYTO	DHC-N01
PCR purification column and extra buffer	Zymo	D4013 and D4004-1-L
Qubit 1× dsDNA HS Assay	Thermo Fisher	Q33230
Bioanalyzer High Sensitivity DNA kit	Agilent	5067-4626

**Oligonucleotides**		

Carrier ssDNA (Table 1)	IDT	N/A
Nextera i5 primers (Table 1)	IDT	N/A
Nextera i7 primers (Table 1)	IDT	N/A

**Software and algorithms**		

Dip-c	(Tan et al., 2018)	https://github.com/tanlongzhi/dip-c
Hickit	(Tan et al., 2018)	https://github.com/lh3/hickit

**Other**		

Dounce homogenizer	Sigma	D8938 (or D9063, D9938, D9188, D0189)
Cell strainer (only necessary for nuclei)	Corning	352340 (or 352235, 352360)
1.5 mL DNA LoBind Tube	Eppendorf	022431021
DNA LoBind 96-well plate	Eppendorf	0030129504 (or 0030129512)
Adhesive film and roller	Bio-Rad	MSB1001 and MSR0001
Flow cytometer	BD	FACSAria
Thermal cycler	Bio-Rad	T100

**Table 1:** Sequences of DNA oligonucleotides

NAME	SEQUENCE
**Carrier ssDNA**	

Carrier ssDNA	TCAGGTTTTCCTGAA

**Nextera i7 Index Primers**	

701	CAAGCAGAAGACGGCATACGAGATTCGCCTTAGTCTCGTGGGCTCGG
702	CAAGCAGAAGACGGCATACGAGATCTAGTACGGTCTCGTGGGCTCGG
703	CAAGCAGAAGACGGCATACGAGATTTCTGCCTGTCTCGTGGGCTCGG
704	CAAGCAGAAGACGGCATACGAGATGCTCAGGAGTCTCGTGGGCTCGG
705	CAAGCAGAAGACGGCATACGAGATAGGAGTCCGTCTCGTGGGCTCGG
706	CAAGCAGAAGACGGCATACGAGATCATGCCTAGTCTCGTGGGCTCGG
707	CAAGCAGAAGACGGCATACGAGATGTAGAGAGGTCTCGTGGGCTCGG
708	CAAGCAGAAGACGGCATACGAGATCCTCTCTGGTCTCGTGGGCTCGG
709	CAAGCAGAAGACGGCATACGAGATAGCGTAGCGTCTCGTGGGCTCGG
710	CAAGCAGAAGACGGCATACGAGATCAGCCTCGGTCTCGTGGGCTCGG
711	CAAGCAGAAGACGGCATACGAGATTGCCTCTTGTCTCGTGGGCTCGG
712	CAAGCAGAAGACGGCATACGAGATTCCTCTACGTCTCGTGGGCTCGG

**Additional Nextera i7 Index Primers (if more than 96 cells need to be pooled)**	

714	CAAGCAGAAGACGGCATACGAGATTCATGAGCGTCTCGTGGGCTCGG
715	CAAGCAGAAGACGGCATACGAGATCCTGAGATGTCTCGTGGGCTCGG
716	CAAGCAGAAGACGGCATACGAGATTAGCGAGTGTCTCGTGGGCTCGG
718	CAAGCAGAAGACGGCATACGAGATGTAGCTCCGTCTCGTGGGCTCGG
719	CAAGCAGAAGACGGCATACGAGATTACTACGCGTCTCGTGGGCTCGG
720	CAAGCAGAAGACGGCATACGAGATAGGCTCCGGTCTCGTGGGCTCGG
721	CAAGCAGAAGACGGCATACGAGATGCAGCGTAGTCTCGTGGGCTCGG
722	CAAGCAGAAGACGGCATACGAGATCTGCGCATGTCTCGTGGGCTCGG
723	CAAGCAGAAGACGGCATACGAGATGAGCGCTAGTCTCGTGGGCTCGG
724	CAAGCAGAAGACGGCATACGAGATCGCTCAGTGTCTCGTGGGCTCGG
726	CAAGCAGAAGACGGCATACGAGATGTCTTAGGGTCTCGTGGGCTCGG
727	CAAGCAGAAGACGGCATACGAGATACTGATCGGTCTCGTGGGCTCGG

**Nextera i5 Index Primers**	

501	AATGATACGGCGACCACCGAGATCTACACTAGATCGCTCGTCGGCAGCGTC
502	AATGATACGGCGACCACCGAGATCTACACCTCTCTATTCGTCGGCAGCGTC
503	AATGATACGGCGACCACCGAGATCTACACTATCCTCTTCGTCGGCAGCGTC
504	AATGATACGGCGACCACCGAGATCTACACAGAGTAGATCGTCGGCAGCGTC
505	AATGATACGGCGACCACCGAGATCTACACGTAAGGAGTCGTCGGCAGCGTC
506	AATGATACGGCGACCACCGAGATCTACACACTGCATATCGTCGGCAGCGTC
507	AATGATACGGCGACCACCGAGATCTACACAAGGAGTATCGTCGGCAGCGTC
508	AATGATACGGCGACCACCGAGATCTACACCTAAGCCTTCGTCGGCAGCGTC

**Additional Nextera i5 Index Primers (if more than 96 cells need to be pooled)**	

510	AATGATACGGCGACCACCGAGATCTACACCGTCTAATTCGTCGGCAGCGTC
511	AATGATACGGCGACCACCGAGATCTACACTCTCTCCGTCGTCGGCAGCGTC
513	AATGATACGGCGACCACCGAGATCTACACTCGACTAGTCGTCGGCAGCGTC
515	AATGATACGGCGACCACCGAGATCTACACTTCTAGCTTCGTCGGCAGCGTC
516	AATGATACGGCGACCACCGAGATCTACACCCTAGAGTTCGTCGGCAGCGTC
517	AATGATACGGCGACCACCGAGATCTACACGCGTAAGATCGTCGGCAGCGTC
518	AATGATACGGCGACCACCGAGATCTACACCTATTAAGTCGTCGGCAGCGTC
520	AATGATACGGCGACCACCGAGATCTACACAAGGCTATTCGTCGGCAGCGTC

All DNA Oligonucleotides were ordered from IDT with standard desalting.

## Materials and equipment

### Reagents to prepare for isolation of nuclei from the brain

• **1.5 M sucrose.** Dissolve 20.538 g sucrose (Sigma 84097) in water and adjust volume to 40 mL. Filter and store indefinitely at 4°C. For long-term storage, aliquot and store indefinitely at −20°C to avoid bacterial growth.

***Note:*** If used regularly, **1.5 M sucrose** may develop white bacterial growth after a few months at 4°C. If so, discard and use another unopened aliquot.

• **Nuclei Isolation Medium 1.** Each reaction consumes 6 mL. The following recipe (45 mL) is sufficient for 7 reactions:

**Table:** 

Reagent	Final concentration	Amount
Water	n/a	36.2625 mL
**1.5 M sucrose**	250 mM (8.56% w/v)	7.5 mL
2 M KCl (ThermoFisher AM9640G)	25 mM	562.5 μL
1 M HEPES pH 7.5 (ThermoFisher 15630080)	10 mM	450 μL
1 M MgCl_2_ (ThermoFisher AM9530G)	5 mM	225 μL
**Total**	**n/a**	**45 mL**

Vortex to mix. Store indefinitely at 4°C.

***Optional:*** If using cells rather than nuclei, reagents to prepare for chromatin conformation capture:

a **10% Igepal CA 630**. Each reaction consumes 20 μL. The following recipe (1 mL) is sufficient for 45 reactions:

**Table:** 

Reagent	Final concentration	Amount
Water	n/a	900 μL
Igepal CA 630 (Sigma I8896)	10%	100 μL
**Total**	**n/a**	**1 mL**

Vortex to mix. Store indefinitely at 18°C–27°C.

### Reagents to prepare for whole-genome amplification

• **60 mg/mL Qiagen Protease.** Dissolve 1 vial (7.5 AU) of Qiagen Protease (Qiagen 19155) in 2.78 mL water. Filter, aliquot, and store indefinitely at 4°C.

***Note:*** If used regularly, **60 mg/mL Qiagen Protease** may develop white precipitates after a few months. If so, discard and use another unopened aliquot.

• **14.3 mM (5 mg/mL) DAPI.** Dissolve 1 tube (10 mg) of DAPI (ThermoFisher D1306) in 2 mL water. Aliquot and store indefinitely at 4°C.
• **0.1 X TE.** The following recipe is for 40 mL:

**Table:** 

Reagent	Final concentration	Amount
Water	n/a	36 mL
TE (ThermoFisher AM9849)	0.1 X	4 mL
**Total**	**n/a**	**40 mL**

Vortex to mix. Store indefinitely at 18°C–27°C.

• **100 μM Carrier ssDNA.** Dissolve each 1 nmol Carrier ssDNA (Table 1; IDT; standard desalting) in 10 μL **0.1 X TE** to a final concentration of 100 μM. Store indefinitely at −20°C.
• **12.5 μM Nextera i5 and i7 Primers.** Dissolve each 1 nmol Nextera i5 or i7 Primer (Table 1; IDT; standard desalting) in 80 μL **0.1 X TE** to a final concentration of 12.5 μM. Store indefinitely at −20°C.

## Step-by-step method details

### Isolation and fixation of nuclei from the human or mouse brain

**Timing: 2 h**

In this step, cell nuclei are isolated from the human or mouse brain through mechanical homogenization (Dounce homogenizer) in the presence of a detergent (0.1% Triton X-100), and preserved with a fixative (2% PFA).

Isolation of nuclei was adapted from (Krishnaswami et al., 2016; Lacar et al., 2016) with minor modifications. In particular, Tris buffer was replaced with an equal molarity of HEPES buffer to avoid interference with PFA fixation.

Note that nuclei isolation may alter native 3D genome structure, because the cytoskeleton and gene transcription may be disrupted. Nuclei should be isolated as fast as possible and kept at 4°C until fixation to minimize changes to the 3D genome. We have not tested fixation before nuclei isolation, because homogenization is generally more challenging for fixed tissues.

#### Nuclei isolation

***Optional:*** If using cells rather than nuclei, skip this section and proceed directly to Fixation (**Step 14**).

1. Chill a 2 mL Dounce homogenizer (Sigma D8938) on ice for up to 200 mg of tissue. For larger tissues, use a homogenizer of a larger size (D9063 for 7 mL, D9938 for 15 mL, D9188 for 40 mL, D0189 for 100 mL) and scale up the reaction accordingly.
2. Freshly prepare **1 mM DTT.** Each reaction consumes 6 μL. The following recipe (1 mL) is sufficient for 150 reactions:

**Table:** 

Reagent	Final concentration	Amount
Water	n/a	1 mL
1 M DTT (aliquoted from Sigma 646563)	1 mM	1 μL
**Total**	**n/a**	**1 mL**

Vortex to mix.

3. Freshly prepare **Nuclei Isolation Buffer without Triton**: The following recipe (6 mL) is for 1 reaction:

**Table:** 

Reagent	Final concentration	Amount
**Nuclei Isolation Medium 1**	n/a	6 mL
**1 mM DTT**	1 μM	6 μL
**Total**	**n/a**	**6 mL**

Vortex to mix. Chill on ice.

4. Freshly prepare **Nuclei Isolation Buffer with Triton**: The following recipe (2 mL) is for 1 reaction:

**Table:** 

Reagent	Final concentration	Amount
**Nuclei Isolation Buffer without Triton**	n/a	2 mL
10% Triton X-100 (Sigma 93443)	0.1%	20 μL
**Total**	**n/a**	**2 mL**

Vortex to mix. Chill on ice.

5. Add 2 mL ice-cold **Nuclei Isolation Buffer with Triton** to the homogenizer.
6. Add up to 200 mg tissue to the homogenizer.
7. Dounce the tissue with 5 strokes of the loose pestle (A), and 15 strokes of the tight pestle (B).
8. Transfer the homogenate to a conical tube.
9. Centrifuge at 100 *g* for 8 min at 4°C.
10. Carefully remove supernatant without disrupting the soft pellet. Resuspend in 2 mL **Nuclei Isolation Buffer without Triton.**
11. Centrifuge at 100 *g* for 8 min at 4°C.
12. Carefully remove supernatant without disrupting the soft pellet. Resuspend in 2 mL **Nuclei Isolation Buffer without Triton.**
13. Filter by a 40-um cell strainer (Corning 352340) or other sizes (352235 for 35 μm, 352360 for 100 μm).

***Note:*** The nuclei suspension may be cloudy because of debris (e.g., myelin). Debris does not affect downstream procedures, and will be partially solubilized during SDS treatment (**Step 26** and **Step 27**) at the Chromatin Conformation Capture step.

#### Fixation

***Optional:*** If using cells rather than nuclei, start from here.

14. Freshly prepare **1% BSA in PBS**: Dissolve 0.1 g BSA (Gemini 700-106P) in 10 mL PBS (ThermoFisher 10010023). Each reaction consumes 1.2 mL. Chill on ice.
15. Add 133.3 μL 32% PFA (EMS 15714; store at 4°C for up to a month after opening) to each 2 mL cells or nuclei (final concentration: 2%).

**CRITICAL:** PFA is hazardous. Perform the above and following steps (until **Step 19**: resuspension of the pellet in **1% BSA in PBS**) in a fume hood and properly dispose of waste.

***Note:*** We have not tested other types of formaldehyde (e.g., methanol-containing), other fixatives, or unfixed cells or nuclei.

16. Rotate at 18°C–27°C for 10 min.
17. Add 200 μL ice-cold **1% BSA in PBS.** Invert to mix.

***Note:*** BSA, rather than the more widely used glycine, is used to react with excess PFA because in our hands, reaction between glycine and PFA acidifies the solution (yellow when phenol red is present, indicating pH < 6) and would dissolve all cells or nuclei if left for too long (> 1 hour on ice).

***Note:*** The above step is not aimed to fully quench PFA. Addition of BSA greatly reduces loss of cells or nuclei by preventing cells or nuclei from sticking to the side of the tube, and from aggregating when spun down and resuspended in **1% BSA in PBS**.

18. Centrifuge at 1000 *g* for 5 min at 4°C.

***Optional:*** If using cells rather than nuclei, centrifuge at 600 *g* instead.

19. Remove supernatant. Resuspend in 1 mL ice-cold **1% BSA in PBS.**

***Note:*** The above step fully quenches PFA, and was adapted from Thomsen et al. (2016).

20. Measure cell or nuclei density with a disposable hemocytometer (INCYTO DHC-N01; manufacturer’s protocol: http://www.incyto.com/shop/item.php?it_id=1482380591) and optionally Trypan Blue (ThermoFisher 15250061) if debris is abundant.
21. Aliquot up to 500 k–1 m cells or nuclei per tube. There is no lower bound in principle; see before you begin for details. Each adult mouse brain approximately corresponds to 8 tubes for the cortex and 2 tubes for the hippocampus (2 sides combined). Too many cells (> a few million) may lead to insufficient digestion/ligation and aggregation of cells or nuclei.
22. Centrifuge at 1000 *g* for 5 min at 4°C.

***Optional:*** If using cells rather than nuclei, centrifuge at 600 *g* instead.

23. Remove supernatant. Store indefinitely at −80°C.

***Note:*** The pellet may be large because of debris. Debris does not affect downstream procedures.

**Pause point:** Fixed cells or nuclei can be stored indefinitely at −80°C.

### Chromatin conformation capture (3C/Hi-C)

**Timing: 2 days (or shorter, if using commercially available kits)**

In this step, after detergent (0.5% SDS) treatment, chromatin in fixed nuclei is digested with restriction enzyme(s) (e.g., MboI, DpnII, and/or NlaIII), and re-ligated with a DNA ligase to form artificial linkages (i.e., “chromatin contacts”) between genomic loci that are far away along the linear sequence but nearby in the 3D space. Success of digestion and ligation is assessed by extracting DNA from a small portion (5%) of the reaction, and measuring its length distribution.

This step was adapted from (Nagano et al., 2017; Rao et al., 2014), and can be replaced with other 3C/Hi-C protocol or commercially available 3C/Hi-C kits such as the Arima-SC kit.

#### Digestion

24. Thaw 500 k–1 m fixed cells or nuclei on ice.
  ***Optional:*** If starting from fixed cells rather than nuclei, perform the following additional steps:
  a. Prepare **Hi-C Lysis Buffer.** Each reaction consumes 1 mL:

**Table:** 

Reagent	Final concentration	Amount
Water	n/a	968 μL
**10% Igepal CA 630**	0.2%	20 μL
1 M Tris pH 8.0 (ThermoFisher AM9855G)	10 mM	10 μL
5 M NaCl (ThermoFisher AM9760G)	10 mM	2 μL
**Total**	**n/a**	**1 mL**

Vortex to mix. Chill on ice.
  b. Freshly prepare **Hi-C Lysis Buffer with Inhibitor.** Each reaction consumes 600 μL:

**Table:** 

Reagent	Final concentration	Amount
**Hi-C Lysis Buffer**	n/a	500 μL
Protease inhibitor (aliquoted from Sigma P8340)	n/a	100 μL
**Total**	**n/a**	**600 μL**

Vortex to mix. Chill on ice.
  c. Resuspend cells in 600 μL ice-cold Hi-C Lysis Buffer with Inhibitor.
  d. Incubate on ice for 15 min, occasionally inverting the tube.
  e. Centrifuge at 2500 *g* for 5 min at 4°C.
  f. Remove supernatant. Resuspend in 500 μL ice-cold **Hi-C Lysis Buffer.**
  g. Centrifuge at 2500 *g* for 5 min at 4°C.

25. Prepare **0.5% SDS.** Each reaction consumes 50 μL. The following recipe (100 μL) is sufficient for 1 reaction:

**Table:** 

Reagent	Final concentration	Amount
Water	n/a	95 μL
10% SDS (Sigma 71736)	0.5%	5 μL
**Total**	**n/a**	**100 μL**

Vortex to mix.

26. Resuspend cells or nuclei in 50 μL **0.5% SDS.**

***Note:*** SDS treatment is necessary to obtain a large number of contacts per cell. Without SDS treatment, the number of contacts per cell may decrease by 2 orders of magnitudes.

27. Incubate at 62°C for 10 min.
28. Add 145 μL water and 25 μL 10% Triton X-100 (Sigma 93443) (final concentration: 1.14%). Pipette to mix.
29. Rotate at 37°C for 15 min.
30. Add restriction enzyme(s) and buffer: 25 μL 10 X NEBuffer 2 (NEB B7002S) and 20 μL 25 U/μL MboI (NEB R0147M). Alternatives include: 25 μL 10 X CutSmart Buffer and 20 μL 10 U/μL NlaIII (NEB R0125L), 25 μL 10 X NEBuffer DpnII and 10 μL 50 U/μL DpnII (NEB R0543M), or a combination of multiple enzymes.
31. Rotate at 37°C for 1–24 h.
32. Take 5% (13 μL out of the total 265 μL) and store at 4°C as a **Digestion Control.**

#### Ligation

33. Centrifuge at 1000 *g* for 5 min at 4°C.
34. Freshly prepare **Ligation Buffer.** Each reaction consumes 2 tubes. The following recipe (1 tube) is sufficient for 0.5 reactions:

**Table:** 

Reagent	Final concentration	Amount
Water	n/a	865 μL
10 X T4 DNA ligase buffer (NEB B0202S)	1 X	100 μL
20 mg/mL BSA (NEB B9000S)	0.1 mg/mL	5 μL
**Total**	**n/a**	**~1 mL**

Vortex to mix.

35. Remove supernatant leaving ∼50 μL. Resuspend in 1 tube of **Ligation Buffer.**
36. Centrifuge at 1000 *g* for 5 min at 4°C.
37. Remove supernatant leaving ∼50 μL. Resuspend in 1 tube of **Ligation Buffer.**
38. Add 10 μL 1 U/μL T4 DNA ligase (ThermoFisher 15224-025). Invert to mix.
39. Incubate at 16°C for 4 h, occasionally inverting to tube.
40. Take 5% (50 μL out of the total 1 mL) and store at 4°C as a **Ligation Control.**
41. Centrifuge at 1000 *g* for 5 min at 4°C.
42. Remove supernatant. Store indefinitely at −80°C.

**Pause point:** Ligated cells or nuclei, as well as the **Digestion Control** and **Ligation Control**, can be stored indefinitely at −80°C.

#### Quality control

43. Centrifuge the **Digestion Control** and **Ligation Control** at 1000 *g* for 5 min at 4°C.
44. Remove supernatant from each control. Add 95 μL PBS (ThermoFisher 10010023) and 5 μL 0.8 U/μL Proteinase K (NEB P8107S) (final concentration: 0.04 U/μL) per control. Vortex to mix.
45. Lyse the controls by running the following PCR program:

**Table:** 

Dip-C QC. Lid temperature: 70°C. Volume: 100 μL			
Step	Temperature	Time	Cycles
Lysis	65°C	1 h	1
Hold	4°C	Forever

46. Purify the controls with PCR purification columns (Zymo D4013; manufacturer’s protocol: https://files.zymoresearch.com/protocols/_d4003t_d4003_d4004_d4013_d4014_dna_clean_concentrator_-5.pdf) using a 1:5 ratio between lysate and DNA Binding Buffer. Elute into 6 μL TE (ThermoFisher AM9849) per control.

***Note:*** For each control (100 μL), add 500 μL DNA Binding Buffer. 6 μL is the minimum elution volume of the column.

**Pause point:** Purified controls can be stored indefinitely at −20°C.

**Figure 1:**
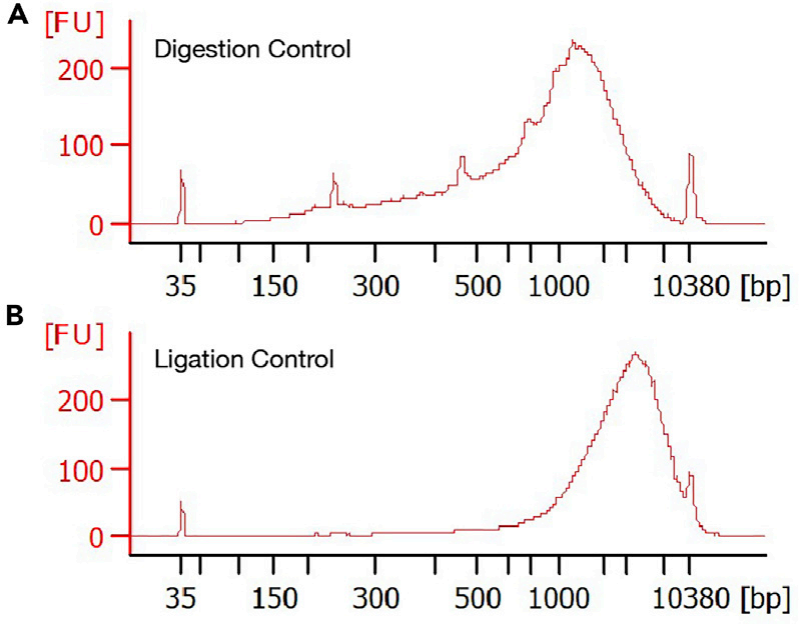
Representative Bioanalyzer traces for quality control of the chromatin conformation capture (3C/Hi-C) step, using a combination of NlaIII and MboI restriction enzymes on mouse cells (A) Digestion Control. (B) Ligation Control. Both were run on a Bioanalyzer High Sensitivity DNA kit.

47. Measure DNA concentration with a Qubit 1× dsDNA HS Assay (ThermoFisher Q33230; manufacturer’s protocol: https://assets.thermofisher.com/TFS-Assets/LSG/manuals/MAN0017455_Qubit_1X_dsDNA_HS_Assay_Kit_UG.pdf). Measure DNA lengths with a Bioanalyzer High Sensitivity DNA kit (manufacturer’s protocol: https://www.agilent.com/cs/library/usermanuals/public/HighSensitivity_DNA_KG.pdf.pdf; or Fragment Analyzer). To evaluate the results, please refer to Expected Outcomes for details, and Figure 1 for representative Bioanalyzer traces.

### Whole-genome amplification (WGA) by tagmentation

**Timing: 1 day**

In this step, single cell or nuclei are sorted into multi-well plates, lysed, and amplified with transposition (Tn5) and PCR.

The procedure below describes amplification with our implementation of the Illumina Nextera chemistry. If higher sensitivity is required—for example, when the number of contacts obtained is insufficient for distinguishing desired cell types or for 3D modeling with desired spatial resolution, please follow the procedure of our multiplex end-tagging amplification (META) method (Tan et al., 2018). META can detect 2 times as many contacts as Nextera, but involves custom Tn5 transposomes and 2 additional PCR steps.

For first-time users, we recommend starting with 1 96-well plate. Experienced users may amplify 4–8 plates at a time, depending on the number of available PCR machines.

Nextera Index Primers listed in the key resources table allow the pooling of up to 384 cells or nuclei to be sequenced on the same lane. Other index designs may allow more cells or nuclei to be pooled (e.g., 10-bp dual Nextera indices from IDT allows 3,840).

#### Flow sorting and lysis

48. Thaw a tube of ligated cells or nuclei on ice.
49. Freshly prepare **300 μM DAPI.** Each reaction consumes 1 μL. The following recipe is sufficient for 100 reactions:

**Table:** 

Reagent	Final concentration	Amount
PBS (ThermoFisher 10010023)	n/a	100 μL
**14.3 mM (5 mg/mL) DAPI**	300 μM	2.1 μL
**Total**	**n/a**	**~100 μL**

Vortex to mix.

50. Resuspend cells or nuclei in 1 mL PBS (ThermoFisher 10010023). Chill on ice.
51. Add 1 μL **300 μM DAPI** (final concentration: 300 nM). Pipette to mix. Transfer to a flow sorting tube and chill on ice.
52. Freshly prepare **Dip-C Lysis Buffer.** Each cell consumes 2 μL. The following recipe (1 mL) is sufficient for 4 96-well plates:

**Table:** 

Reagent	Final concentration	Amount
Water	n/a	929 μL
1 M DTT (aliquoted from Sigma 646563)	25 mM	25 μL
1 M Tris pH 8.0 (ThermoFisher AM9855G)	20 mM	20 μL
10% Triton X-100 (Sigma 93443)	0.15%	15 μL
**100 μM Carrier ssDNA**	500 nM	5 μL
5 M NaCl (ThermoFisher AM9760G)	20 mM	4 μL
0.5 M EDTA (ThermoFisher AM9260G)	1 mM	2 μL
**60 mg/mL Qiagen Protease**	15 μg/mL	0.25 μL
**Total**	**n/a**	**1 mL**

Vortex to mix. Aliquot to 80 μL in 12-strip tubes.

***Note:*** Addition of Carrier ssDNA reduces loss of input DNA materials by preventing genomic DNA from sticking to the side of the tube, especially in PCR tubes that are not low-retention.

***Note:*** Volume (0.25 μL) of **60 mg/mL Qiagen Protease** does not need to be exact. If desired, however, pipetting accuracy can be increased by freshly diluting **60 mg/mL Qiagen Protease** prior to addition (e.g., 1:100 dilution followed by the addition of 25 μL instead of 0.25 μL).

***Optional:*** Before the addition of **60 mg/mL Qiagen Protease**, **Dip-C Lysis Buffer** can be stored indefinitely at −20°C.

53. Add 2 μL **Dip-C Lysis Buffer** per well to a DNA low-bind 96-well plate (semi-skirted: Eppendorf 0030129504; or skirted: Eppendorf 0030129512, depending on the FACS and PCR machines).

***Note:*** To maximize speed, use a 12-channel pipette to add solution to each well in the above and all subsequent steps.

54. Seal with film (Bio-Rad MSB1001) and a roller (Bio-Rad MSR0001).

**Pause point: Dip-C Lysis Buffer** can be stored on ice for a few hours before sorting.

**Figure 2:**
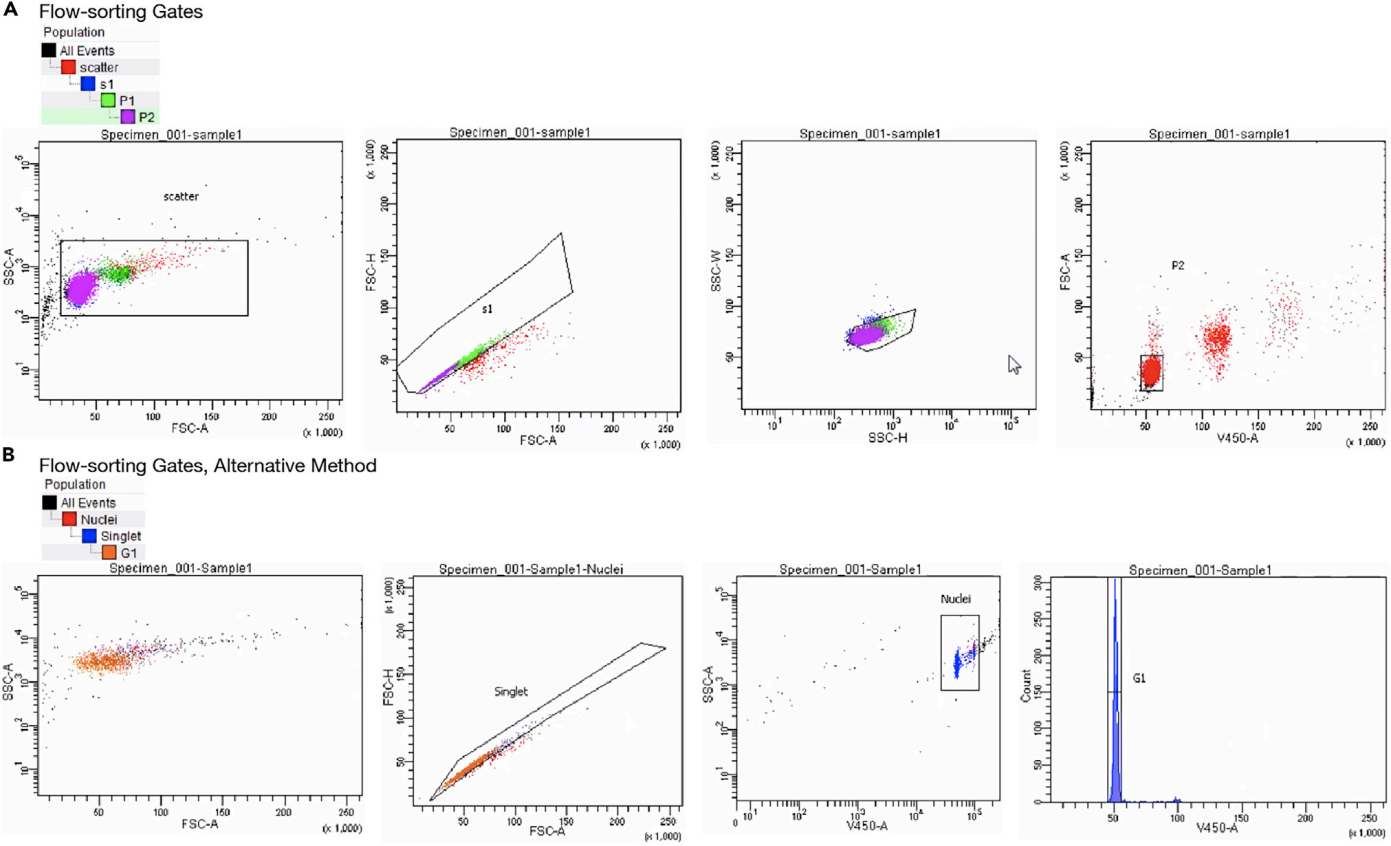
Representative flow cytometry diagrams with 2 roughly equivalent gating strategies The minor fraction of particles with double, triple, or even higher DAPI signals (“V450-A”) were aggregates from the Chromatin Conformation Capture step. Both were run on a BD FACSAria flow sorter. The 2 gating strategies arose from personal preferences of different flow cytometer operators, and do not affect the results. Note that we primarily study cells in the G0/G1 phase of the cell cycle; the corresponding gate (e.g., “G1” in (B)) should be adjusted when studying other phases of the cell cycle.

55. Sort a single diploid cell or nucleus per well, based on DAPI signal (linear scale). Seal tightly to avoid evaporation. Please refer to Expected Outcomes for details, and Figure 2 for representative flow cytometer diagrams and gates.

**CRITICAL:** Once cells or nuclei are sorted into **Dip-C Lysis Buffer**, avoid cross contamination of liquid between wells.

56. Centrifuge at 1000 *g* for 1 min.

**Pause point:** Sorted cells or nuclei in **Dip-C Lysis Buffer** can be stored on ice for a few hours before lysis.

57. Lyse the cells by running the following PCR program:

**Table:** 

Dip-C lysis. Lid temperature: 75°C. Volume: 2 μL			
Step	Temperature	Time	Cycles
Lysis	50°C	1 h	1
Heat inactivation	70°C	15 min	1
Hold	4°C	Forever	

Store at −80°C.

**Pause point:** Lysed cells or nuclei can be stored for a few months at −80°C.

***Note:*** The film (Bio-Rad MSB1001) may peel over time at −80°C. This can be avoided by changing to cold-resistant film (Bio-Rad MSF1001) after lysis.

***Optional:*** For longer-term storage, cells or nuclei can be sorted into empty 96-well plates rather than **Dip-C Lysis Buffer**, and stored indefinitely at −80°C.

#### Transposition

58. Prepare **Transposition Buffer.** Each well consumes 8 μL. The following recipe (1 mL) is sufficient for 1 96-well plate:

**Table:** 

Reagent	Final concentration	Amount
Water	n/a	781.25 μL
50% PEG 8000 (Hampton Research HR2-535)	10%	200 μL
1 M TAPS pH 8.5 (Boston Bio Products BB-2375)	12.5 mM	12.5 μL
1 M MgCl2 (ThermoFisher AM9530G)	6.25 mM	6.25 μL
**Total**	**n/a**	**1 mL**

Vortex to mix.

***Optional:* Transposition Buffer** can be stored indefinitely at −20°C.

59. Freshly prepare **Transposition Mix.** Each well consumes 8 μL. The following recipe is sufficient for 1 96-well plate (with 10% overhead):

**Table:** 

Reagent	Final concentration	Amount
**Transposition Buffer**	n/a	844.8 μL
Nextera Tn5 transposome (homemade at 125 nM (Tan et al., 2018) or TDE1 of Illumina 20034197)	n/a	~1.6 μL
**Total**	**n/a**	**~850 μL**

Pipette to mix. Aliquot to 69 μL in 12-strip tubes.

**Figure 3:**
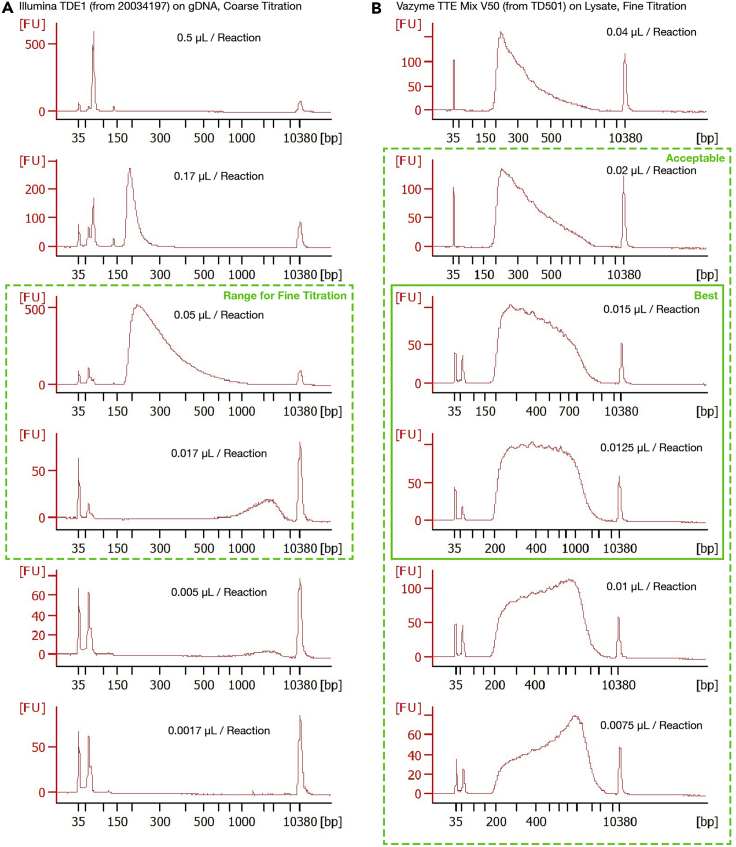
Representative Bioanalyzer traces for titration of Tn5 transposome concentration during the whole-genome amplification (WGA) by tagmentation step (A) Coarse titration of Illumina TDE1 on purified HeLa gDNA. Range for further titration is indicated by a dashed green box. Note that gDNA only gives approximate results because transposition is slightly different between gDNA and lysate. (B) Fine titration of Tn5 transposome from a different vendor (TTE Mix V50 from Vazyme TD501) on nuclei lysate (see troubleshooting 2 for details). Range suitable for sequencing is indicated by a dashed green box ("acceptable”), and the optimal concentration shown by a solid green box (“best”). All were run on a Bioanalyzer High Sensitivity DNA kit.

**CRITICAL:** The amount of Tn5 transposome per well (∼0.015 μL above) determines the length of the final sequencing library. It should be titrated in a pilot experiment with a concentration gradient of Tn5 transposome to obtain an average length of ∼500 bp. Please refer to troubleshooting 2 for details, and Figure 3 for representative Bioanalyzer traces.

***Note:*** Nextera Tn5 transposome is also available from Vazyme (TTE Mix V50 of TD501) and from Diagenode (C01070012; not tested). We primarily use Vazyme.

***Optional:*** For first-time users, a **Positive Control** well can be set up as 2 μL of a 5 pg/μL dilution of any genomic DNA (e.g., diluting 100 ng/μL HeLa gDNA (NEB N4006S) 1:20,000 in water). A **Negative Control** well can be set up as 2 μL water. Please refer to troubleshooting 1 for details.

60. Add 8 μL **Transposition Mix** per well (total volume: 10 μL), avoiding touching the liquid (i.e., pipette onto the side, rather than the bottom, of the well). Vortex and spin down.

***Note:*** Before PCR amplification, we typically avoid touching the liquid with pipette tips to minimize loss of input DNA materials. In particular, if pipette tips touch the liquid, genomic DNA may stick to the tips and get lost when tips are withdrawn from the liquid. However, the efficacy of this precaution has not been tested systematically; touching the liquid may be acceptable if the resulting data is satisfactory.

61. Transpose the genome by running the following PCR program:

**Table:** 

Dip-C transposition. Lid temperature: 60°C. Volume: 10 μL			
Step	Temperature	Time	Cycles
Transposition	55°C	10 min	1
Hold	4°C	Forever

#### Stopping

62. Freshly prepare **Stop Mix.** Each well consumes 2 μL. The following recipe (1 mL) is sufficient for 4 96-well plates:

**Table:** 

Reagent	Final concentration	Amount
Water	n/a	849 μL
0.5 M EDTA (ThermoFisher AM9260G)	45 mM	90 μL
5 M NaCl (ThermoFisher AM9760G)	300 mM	60 μL
10% Triton X-100 (Sigma 93443)	0.01%	1 μL
**60 mg/mL Qiagen Protease**	100 ug/mL	1.667 μL
**Total**	**n/a**	**1 mL**

Vortex to mix. Aliquot to 80 μL in 12-strip tubes.

***Optional:*** Before the addition of **60 mg/mL Qiagen Protease**, **Stop Mix** can be stored indefinitely at −20°C.

***Note:*** Addition of 10% Triton X-100 is for ease of pipetting.

63. Add 2 μL **Stop Mix** per well (total volume: 12 μL per well), avoiding touching the liquid (i.e., pipette onto the side, rather than the bottom, of the well). Vortex and spin down.
64. Stop transposition by running the following PCR program:

**Table:** 

Dip-C Stop. Lid temperature: 75°C. Volume: 12 μL			
Step	Temperature	Time	Cycles
Removal of Tn5 transposase	50°C	40 min	1
Heat inactivation	70°C	20 min	1
Hold	4°C	Forever

**Pause point:** Stopped reactions can be stored on ice for a few hours before amplification.

#### Amplification

65. Freshly prepare **PCR Mix.** Each well consumes 11 μL. The following recipe (1.178 mL) is sufficient for 1 96-well plate (with 10% overhead):

**Table:** 

Reagent	Final concentration	Amount
Q5 Reaction Buffer (NEB M0491S)	n/a	528 μL
Q5 High GC Enhancer (NEB M0491S)	n/a	528 μL
10 mM (each) dNTP mix (NEB N0447S)	538 μM (each)	63.36 μL
1 M MgCl2 (ThermoFisher AM9530G)	5.38 mM	6.336 μL
20 mg/mL BSA (NEB B9000S)	448 ug/mL	26.4 μL
2 U/μL Q5 DNA Polymerase (NEB M0491S)	0.0448 U/μL	26.4 μL
**Total**	**n/a**	**1.178 mL**

Vortex to mix. Aliquot to 97 μL in 12-strip tubes.

66. Add 1 μL **12.5 μM Nextera i5 Primer** and 1 μL **12.5 μM Nextera i7 Primer** per well (total volume: 14 μL per well; final concentration during PCR: 500 nM each), avoiding touching the liquid (i.e., pipette onto the side, rather than the bottom, of the well). Arrange the indices so no cells share the same index on each sequencing run; see Figure 4 for an example arrangement.
  ***Optional:*** For simpler pipetting, **12.5 μM Nextera i5 Primer** and **12.5 μM Nextera i7 Primer** can be 1:1 pre-mixed into a 96-well plate (6.25 μM each), and stored indefinitely at −20°C.

**Figure 4:**
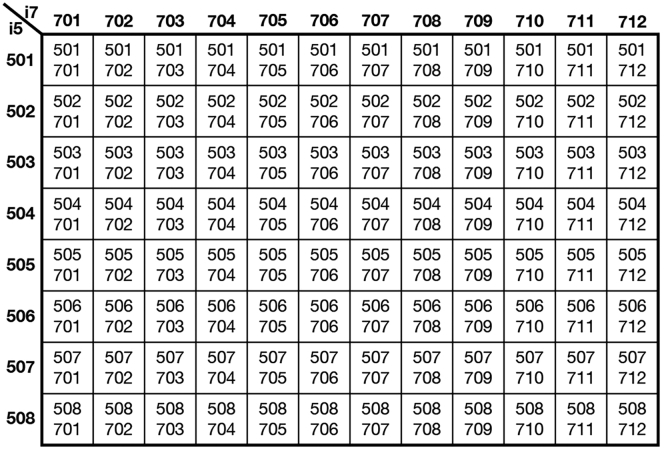
Example arrangement of Nextera i7 and i5 indices on a 96-well plate
67. Add 11 μL **PCR Mix** per well (total volume: 25 μL per well), avoiding touching the liquid (i.e., pipette onto the side, rather than the bottom, of the well). Vortex and spin down.

68. Amplify the genome by running the following PCR program:

**Table:** 

Dip-C PCR. Lid temperature: 105°C. Volume: 25 μL			
Step	Temperature	Time	Cycles
Preheating lid	4°C	3 min	1
Filling in gap	72°C	3 min	1
Initial denaturation	98°C	20 s	1
Denaturation	98°C	10 s	14
Annealing	62°C	1 min	
Extension	72°C	2 min	
Final extension	72°C	5 min	1
Hold	4°C	Forever

***Note:*** The above PCR program consists of 14 cycles, which is suitable for the input DNA amount of human and mouse samples (∼6 pg per cell or nucleus, given a diploid genome size of ∼6 Gb). The number of cycles may need adjustment if an organism has a very different genome size.

**Pause point:** PCR reactions can be stored on ice for a few hours, or indefinitely at −20°C.

#### Purification and size selection

69. Pool all wells from a 96-well plate.
70. Purify with PCR purification columns (Zymo D4013) using a 1:5 ratio between PCR reaction and DNA Binding Buffer (Zymo D4004-1-L to order extra). Elute into 400 μL TE (ThermoFisher AM9849) per plate.

**CRITICAL:** Avoid cross contamination of liquid between plates that use overlapping indices.

***Note:*** Each 96-well plate (total volume: 2.4 mL) can be pooled directly into 12 mL DNA Binding Buffer and vortexed (total volume: 14.4 mL). Because each PCR purification column can only load 800 μL at a time, we typically use 6 columns per plate to save time; each column only needs to be loaded 3 times. After loading and washing, elute each column into 66.7 μL TE (ThermoFisher AM9849) and pool (total volume: 400 μL).

***Optional:*** For the **Positive Control** and **Negative Control**, each well (25 μL) is mixed with 125 μL DNA Binding Buffer. Elute each into 6 μL TE (ThermoFisher AM9849).

**Pause point:** Purified libraries can be stored indefinitely at −20°C.

**Figure 5:**
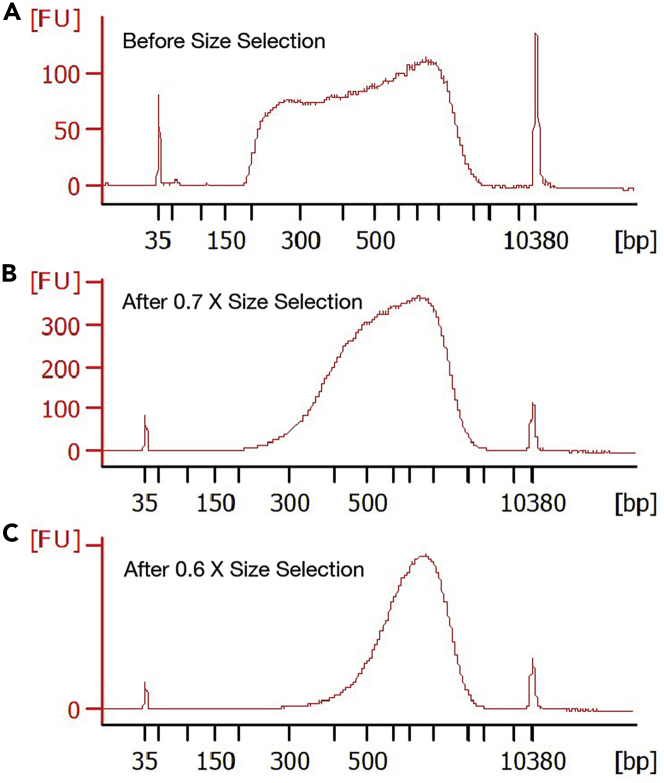
Representative Bioanalyzer traces before and after size selection of a sequencing library (A) Before size selection. (B) After size selection with 0.7 X SPRISelect beads. (C) Similar to (B) but with 0.6 X beads. All were run on a Bioanalyzer High Sensitivity DNA kit.
72. Remove short fragments from half of the library (200 μL) with 0.7 X (140 μL) or 0.6 X (120 μL) SPRIselect beads (Beckman Coulter B23317; manufacturer’s protocol: https://www.beckman.com/techdocs/B24965AA/wsr-128718). Elute into 50 μL TE (ThermoFisher AM9849).

***Note:*** The remaining half (∼200 μL) serves as a back-up in case size selection or sequencing fails. Depending on sample submission requirements (i.e., minimum DNA amount) of the sequencing provider, the above purification and size selection steps can be scaled down (e.g., to half of the volume), with any extra liquid stored indefinitely at −20°C in the form of a mixture of PCR reactions and DNA Binding Buffer.

71. Measure DNA concentration with a Qubit 1× dsDNA HS Assay. Measure DNA lengths with a Bioanalyzer High Sensitivity DNA kit (or Fragment Analyzer). To evaluate the results, please refer to Expected Outcomes for details, and Figure 3 and 5A for representative Bioanalyzer traces.

**Pause point:** Final libraries can be stored indefinitely at −20°C.

73. Measure DNA concentration with a Qubit 1× dsDNA HS Assay. Measure DNA lengths with a Bioanalyzer High Sensitivity DNA kit (or Fragment Analyzer). To evaluate the results, please refer to Expected Outcomes for details and Figure 5 for representative Bioanalyzer traces.

#### Sequencing and data analysis

74. Sequence on an Illumina sequencer (e.g., HiSeq or NovaSeq) with paired-end 150-bp reads and dual 8-bp indices.

***Note:*** To saturate the sequencing library, we sequence each cell with 3–6 m read pairs.

***Note:*** If 3D reconstruction of diploid genome structures (by reading heterozygous SNPs) is not required, shorter read lengths (e.g., paired-end 75 bp) can be used.

***Optional:*** Before deep sequencing, the presence and prevalence of chromatin contacts can be tested at almost no cost with as few as 1,000 reads per plate (e.g., on a MiSeq), which allows the calculation of the “contact rate” ***CR*** and the “contact density” ***CD***; see quantification and statistical analysis for details.

75. Analyze data with the dip-c (https://github.com/tanlongzhi/dip-c) package.

## Expected outcomes

For isolation and fixation of cell nuclei, we typically obtain 6 m fixed nuclei from the mouse cortex, 1.5 m from the mouse hippocampus (2 sides combined), 40 m from the mouse cerebellum, 2 m from 100 mg human cortex, and 40 m from 100 mg human cerebellum.

For chromatin conformation capture (3C/Hi-C), the **Digestion Control** should have a typical length of 2 kb on a Bioanalyzer High Sensitivity DNA chip, whereas the **Ligation Control** should have a peak around the upper marker (10 kb) (Figure 1). If starting from 500 k cells or nuclei (i.e., 500 k × 6.6 pg = 3 ug genomic DNA), each control (5% of the reaction, eluted into 6 μL) should yield a concentration of 30 ng/μL.

For flow-sorting, single cell or nuclei should be easily distinguished based on DAPI signal (on a linear scale) from debris (no signal) and from clumps of multiple cells or nuclei (double, triple, or even higher signal; should be a minority of events) (Figure 2). We additionally use a typical “singlet” gate based on FSC signal height (or width on some sorters).

For whole-genome amplification (WGA), the Tn5 concentration (∼0.015 μL per cell) should be titrated so that the PCR product (before size selection) has a relatively flat—slightly higher on the shorter (left) side—length distribution on a Bioanalyzer High Sensitivity DNA chip (i.e., an average length of 500 bp) (Figures 3 and 5A). We typically obtain a concentration of 4 ng/μL both before (eluted into 400 μL per 96-well plate) and after (eluted into 50 μL from half a reaction of a 96-well plate) size selection (Figure 5).

## Quantification and statistical analysis

For general diagnosis of whole-genome amplification (WGA)—e.g., mapping rate, genome coverage, and library complexity, please refer to relevant literature (Chen et al., 2017; Huang et al., 2015) for guidelines.

Success of Dip-C in each cell can be assessed by calculating

the “contact rate” (the percentage of read pairs that harbor contacts) ***CR*** = ***C*** / ***R*** × 100%, where

***C*** = the number of contacts,

***R*** = the number of read pairs.

Note that ***CR*** can be calculated either from numbers before deduplication—i.e., ***C***_***raw***_ and ***R***_***raw***_, or from numbers after deduplication—i.e., ***C***_***dedup***_ and ***R***_***dedup***_. The 2 methods are roughly equivalent because the bias of WGA and sequencing and the distribution of contacts are relatively uniform across the genome and independent of each other. For convenience, we typically use the former.

A successful reaction should lead to ***CR*** ≥ 3% (ideally, ≥ 5%). More aggressive size selection (e.g., 0.6 X rather than 0.7 X) will yield a higher ***CR*** (therefore costing less per contact), but at the expense of a lower ***C***_***dedup***_ (because contacts that are located in shorter fragments are lost during size selection).

***CR*** depends on the length distribution of the final sequencing library, because longer reads are more likely to contain contacts. A metric that depends only on the chromatin conformation capture (3C/Hi-C) step (i.e., independent of WGA) is

the “contact density” (the average number of contacts per base pair (bp) of genome) ***CD*** = ***CR*** / ***L***, where

***L*** = the average insert size of the sequenced library (in base pairs (bp)).

In particular, because ***CR*** is the number of contacts per read pair (i.e., the fraction of contact-containing read pairs among all read pairs) and ***L*** is the number of bp per read pair, the formula ***CD* = *CR* / *L*** gives the number of contacts per bp—i.e., the contact density. Note that ***L*** should be calculated from alignments of read pairs that do not harbor contacts—i.e., proper read pairs—rather than from Bioanalyzer traces, because sequencers preferentially read shorter fragments.

For a typical Dip-C reaction, ***CR*** = 10%, ***L*** = 250 bp, leading to ***CD*** = 4 × 10^–4^ /bp = 1 / (2.5 kb); in other words, a typical chromatin conformation capture reaction generates 1 contact every ∼2.5 kb along the genome.

The degree of sequencing saturation can be assessed by calculating

the “duplication rate” (the percentage of contacts that are duplicates) ***DR*** = (1 – ***C***_***dedup***_ / ***C***_***raw***_) × 100%.

A cell is sequenced to saturation (i.e., sequencing deeper will not lead to many more contacts) if ***DR*** ≥ 70% (i.e., on average, each contact is sequenced 3 times). With Nextera amplification, saturation typically occurs with 3–6 m read pairs per cell (i.e., 1 or 2 HiSeq lanes per 96-well plate). At saturation, we typically obtain ***C***_***dedup***_ = 400 k contacts per cell with Nextera, and ***C***_***dedup***_ = 1 m with META.

Note that sequencing to saturation is not necessary for certain analysis. For example, clustering of cell types based on single-cell chromatin A/B compartment values (scA/B) may work with as few as 20–50 k contacts per cell, depending on the cell types (Tan et al., 2021; Tan et al., 2019).

**Figure 6:**
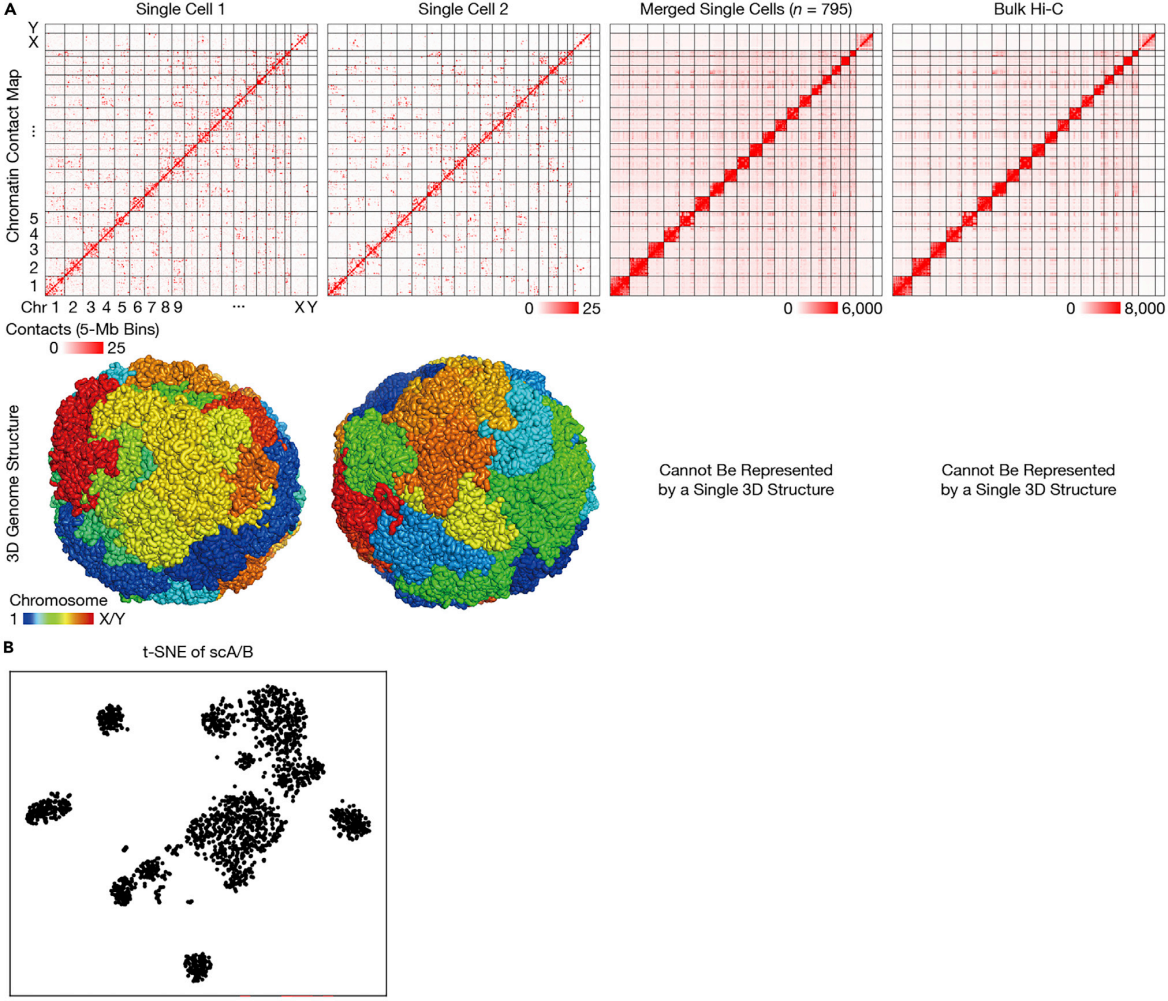
Representative data from the mouse brain (A) Chromatin contact maps (top) and 3D genome structures (bottom) of 2 representative single cells, an aggregation of 795 single cells, and bulk Hi-C. All samples were adult neurons from the mouse brain (Tan et al., 2021). Unlike bulk Hi-C, single-cell contact maps show a characteristic pattern of random “patchiness”—especially for inter-chromosomal contacts—indicating highly heterogenous chromosome interactions among single cells (e.g., each chromosome territory only borders a few others in each cell). Raw bulk Hi-C data was downloaded from (Jiang et al., 2017) and reanalyzed by (Tan et al., 2021). Contact maps were visualized with Juicebox.js (Robinson et al., 2018). Note that aggregated or bullk data cannot be represented by a single 3D genome structure, because such data contain mutually conflicting contacts (e.g., inter-chromosomal contacts between all pairs of chromosomes) that is physically impossible for a single structure. (B) t-SNE plot of scA/B showing clusters of 3D genome structure types, from the mouse brain.

When visualized in Juicebox.js (Robinson et al., 2018), single-cell contact maps should exhibit strong diagonal blocks for intra-chromosomal contacts (same as in bulk Hi-C), and “patchy” off-diagonal blobs for inter-chromosomal contacts that are different among cells (a phenomenon unique to single cells). Figure 6A shows contact maps and 3D reconstructions from representative mouse brain cells in comparison with bulk Hi-C; Figure 6B shows a representative t-SNE plot of scA/B (the first 20 principal components) from ∼2,000 mouse brain cells.

## Limitations

The majority of Dip-C data analysis—including the generation of single-cell chromatin contact maps, calculation of the scA/B matrix, and clustering and identification of cell types—can be performed on any samples. However, reconstruction of 3D structures is limited to normal diploid (requiring a phased SNP file) or haploid cells. In particular, genomic regions with more than 2 copies (i.e., copy number gain) or with 2 identical copies (i.e., loss of heterozygosity (LOH)) cannot be 3D reconstructed.

## Troubleshooting

### Problem 1

No amplification product (i.e., nearly 0 ng/μL DNA concentration at step 71) after PCR purification.

### Potential solution

If the **Positive Control** (pure genomic DNA) also failed to amplify, check reagents that are crucial for whole-genome amplification. For example, sufficient MgCl_2_ must be present in the **Transposition Buffer** (step 58) and in the **PCR Mix** (step 65).

If the **Positive Control** amplified but cells or nuclei did not, check reagents that are crucial for lysis. For example, Qiagen Protease must be present in the **Dip-C Lysis Buffer** (step 52). Alternatively, check the flow-sorting procedure (e.g., alignment of the plate) to ensure cells or nuclei are sorted into each well (step 55). Up to 100 cells or nuclei can be sorted or pipetted into a control well for diagnosis.

### Problem 2

DNA length is too short or too long (at step 71) after PCR purification.

### Potential solution

If DNA length is too short, decrease the concentration of Tn5 transposome (step 59). If decreasing Tn5 does not work, decrease the concentration of Qiagen Protease in the **Dip-C Lysis Buffer** (step 52, and/or the **Stop Mix**) to combat lot variation in Qiagen Protease. If DNA is too long, perform the opposite adjustment.

For first-time users, the Tn5 concentration can be roughly titrated with the **Positive Control** (pure genomic DNA) for maximum DNA yield, and finely titrated with cells or nuclei to obtain the desired DNA length (i.e., 500 bp on average) (Figure 3).

If flow-sorting single cells or nuclei is challenging for titration, a large number of cells or nuclei can be lysed together in the **Dip-C Lysis Buffer**. The aliquoted lysate (stored indefinitely at −80°C) can be used in place of single cells or nuclei for convenient titration (Figure 3B).

### Problem 3

After sequencing (step 74), some of the 96 wells on a plate yield very few read pairs.

### Potential solution

Check the flow-sorting procedure (e.g., alignment of the plate) to ensure cells or nuclei are sorted into each well (step 55). If the plate was not aligned, the flow sorter may miss entire rows or columns of wells.

### Problem 4

After sequencing, the contact rate ***CR*** is too low (< 3%) (at step 75).

### Potential solution

A low ***CR*** indicates that despite successful whole-genome amplification, the chromatin conformation capture step did not generate sufficient contacts. Typically, ***CR*** and ***CD*** are relatively uniform among cells or nuclei from the same 3C/Hi-C reaction, and vary mostly between reactions and/or cell types.

Check the **Digestion Control** and **Ligation Control** (step 47)—the problem is typically caused by insufficient digestion. Switch to another restriction enzyme (e.g., NlaIII usually leads to better digestion), a combination of restriction enzymes, or another 3C/Hi-C protocol or kit. Alternatively, redoing the experiment may lead to a better batch. It’s common in 3C/Hi-C to sequence multiple replicates shallowly (step 74), and proceed with those with higher ***CR***.

Note that certain cell types may be especially challenging. For example, rod photoreceptors (Tan et al., 2019) yield fewer contacts per cell, whereas sperm yield almost no contacts per cell.

### Problem 5

After 3D modeling (Step 75), 3D genome structures have poor quality (e.g., high root-mean-square deviation (RMSD) between replicate structures).

### Potential solution

If possible, obtain more contacts per cell. For example, if the library has not been sequenced to saturation (quantification and statistical analysis; duplication rate ***DR*** < 70%), sequence deeper. Alternatively, switch to another restriction enzyme (e.g., NlaIII usually leads to better digestion), a combination of restriction enzymes, or another 3C/Hi-C protocol or kit to increase the contact rate ***CR***. Switch to META from Nextera to detect more contacts.

Alternatively, reduce the resolution of 3D modeling (e.g., from 20 kb to 100 kb). Fewer contacts are required to determine a lower-resolution structure.

Note that certain cell types and chromosome configurations may be especially challenging for existing 3D-modeling algorithms. 3D modeling (e.g., the choice of energy function and energy-minimization procedure) is an area of active research. For example, cells with more inter-chromosomal contacts tend to be easier to model (Stevens et al., 2017). Cells with complex nuclear shapes (e.g., rings, multiple lobes) may yield high RMSD. For diploid cells, low SNP density (e.g., female DBA/2J mice has very few heterozygous SNPs on Chr X) and homolog interactions (e.g., the 2 copies of human Chr 19 both prefer the nuclear center, and may thus interact by chance) may lead to poor 3D modeling of certain regions.

## Resource availability

### Lead contact

Further information and requests for resources and reagents should be directed to and will be fulfilled by the lead contact, Longzhi Tan (tttt@stanford.edu).

### Materials availability

This study did not generate new unique reagents.

### Data and code availability

The code generated during this study is available at GitHub (https://github.com/tanlongzhi/dip-c).
